# The Use of Bovine Xenogeneic Bone Graft for Dega Pelvic Osteotomy in Children with Hip Dysplasia: A Retrospective Study of 147 Treated Hips

**DOI:** 10.3390/jcm9072241

**Published:** 2020-07-15

**Authors:** Norbert Stiel, Menard Moritz, Kornelia Babin, Anna Suling, Martin Rupprecht, Frank T. Beil, Ralf Stuecker, Alexander S. Spiro

**Affiliations:** 1 Department of Pediatric Orthopaedics, Altonaer Children’s Hospital, 22763 Hamburg, Germany; norbert.stiel@kinderkrankenhaus.net (N.S.); menard.moritz@kinderkrankenhaus.net (M.M.); martin.rupprecht@kinderkrankenhaus.net (M.R.); ralf.stuecker@kinderkrankenhaus.net (R.S.); 2 Department of Pediatric Orthopaedics, Schoen Clinic Hamburg Eilbek, 22081 Hamburg, Germany; KBabin@schoen-klinik.de; 3 Department of Medical Biometry and Epidemiology, University Medical Center Hamburg-Eppendorf, 20251 Hamburg, Germany; asuling@uke.de; 4 Department of Orthopaedics, University Medical Center Hamburg-Eppendorf, 20251 Hamburg, Germany; ft.beil@uke.de

**Keywords:** dega osteotomy, xenograft, children, hip dysplasia

## Abstract

Backgrounds: Dega pelvic osteotomy is commonly used to correct acetabular dysplasia in children with open triradiate cartilage. The use of bovine xenogeneic bone graft (Tutobone^®^) for Dega osteotomy has not been reported so far. This study aimed to determine the clinical and radiological outcome in a large series of children with hip dysplasia who were treated by Dega osteotomy using a bovine xenogeneic block for stabilisation. Methods: A retrospective, single-centre study was conducted including 101 patients (147 hips) with different underlying diseases. The acetabular angle of Hilgenreiner (AA) and the lateral center-edge angle (LCA) were analysed to quantify the correction of acetabular indices. Graft incorporation was assessed using the Goldberg scoring system. Results: the mean preoperative AA improved from 28.1 (SD: 6.7) to 14.7 (SD: 5.1) after surgery (*p* < 0.001). The mean preoperative LCA improved from 9.9 (SD: 6.7) to 21.8 (SD: 6.8) postoperatively (*p* < 0.001). Both indices remained stable at the one-year follow-up examination. Graft incorporation was excellent with a mean Goldberg score of 6.6. Heterotopic ossification occurred in one hip without clinical relevance. Graft-related complications were not noted. Conclusions: Dega osteotomy using Tutobone^®^ is safe and effective in the treatment of acetabular dysplasia in children independent of the underlying disease.

## 1. Introduction

The treatment of hip dysplasia is still challenging. This condition may lead to pain, immobilisation and the development of hip arthritis, if left untreated [1,2]. Different types of surgical procedures exist to correct acetabular dysplasia in children with open triradiate cartilage. Dega osteotomy is one of the most common. The iliac osteotomy introduced by Wiktor Dega is an acetabuloplasty that allows correction of anterior, posterior and especially lateral deficiencies of acetabular coverage [3]. As interposition material for Dega osteotomy autogenous iliac crest or femoral autograft is frequently used. A solid graft composition is important to maintain correction and avoid collapse of the graft [4]. Especially in patients with cerebral palsy osteoporotic or osteopenic bone is commonly found around the hip joint, which may result in decreased strength of the harvested autografts [5]. The use of autogenous iliac crest bone graft may lead to growth disturbances caused by splitting of the iliac apophysis. Moreover, increased blood loss and an increased operative time are reported [6,7]. Further problems include infection, scar tenderness, hernia, persistent pain and paraesthesia [8,9,10]. To avoid these negative side effects many surgeons prefer bone graft substitutes for Dega osteotomy. Different types of bone substitutes exist with varying degrees of osteoconductive and osteoinductive properties. Tutobone^®^ (Tutogen Medical GmbH, Neunkirchen am Brand, Germany) is a bovine cancellous bone graft, which is used as a graft material for Dega osteotomy at our institution for several years. The outcome after Dega pelvic osteotomy using Tutobone^®^ has been reported in one previous study only with a limited number of included cases (12 treated hips) [11]. This study aimed to determine the clinical and radiological outcome in a large series of children with hip dysplasia who were treated by Dega pelvic osteotomy using a Tutobone^®^ block for stabilisation. Special attention was given to quantify the correction and change of acetabular indices over time and to analyse bone graft incorporation.

## 2. Materials and Methods 

### 2.1. Bovine Xenogeneic Bone Graft

Tutobone^®^ is a biocompatible bovine cancellous bone graft, which is widely available. It has osteoconductive properties and is similar to natural human bone owing to its porosity and biomechanical characteristics [12]. The immunogenic potential is minimized, due to an elution and sterilisation process prior to application, but the stability is preserved [13]. 

### 2.2. Patients

The medical records and radiographs of all patients with hip dysplasia treated by Dega pelvic osteotomy using a Tutobone^®^ block between 2012 and 2018 at a single institution were reviewed. Radiographs and clinical data were registered in an electronic database since 2012 at our institution. Previous data were not evaluated for the purpose of this study in order to avoid potential measurement bias (poor image quality in some cases; manual measurement of acetabular angle on printed X-rays). The indication for surgery was persistent acetabular dysplasia in children with open triradiate cartilage. Inclusion criteria were defined as followed: children with Dega pelvic osteotomy using a Tutobone^®^ block for stabilisation (1), and availability of radiographs and clinical data prior to surgery as well as during the first week after surgery, at 6 weeks after surgery and after at least 8 months after surgery in each case (2). Exclusion criteria were defined as followed: loss to follow-up, incomplete radiographs or clinical data, and previous pelvic osteotomy. 

### 2.3. Surgical Procedure and Perioperative Care

**Figure 1:**
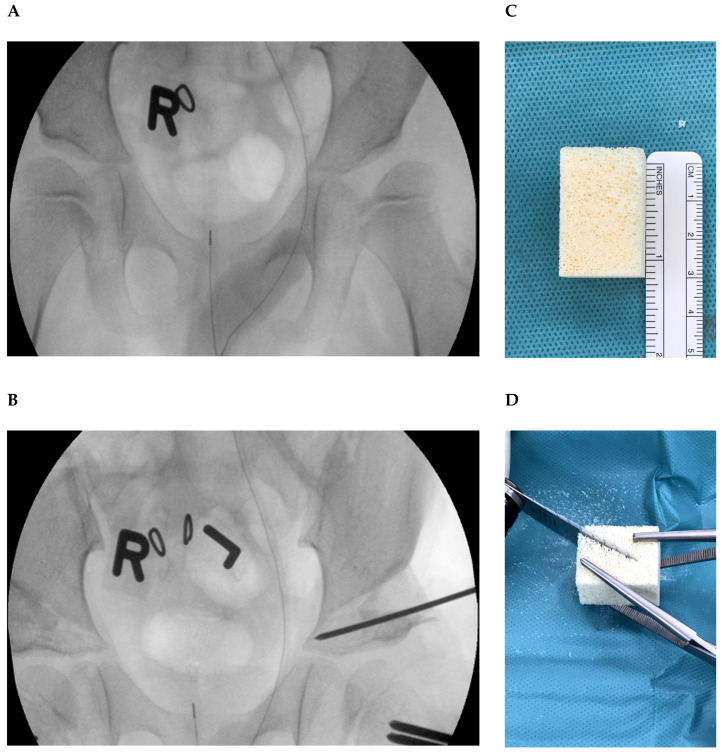
Intraoperative anteroposterior radiograph of the pelvis of a 7-years old boy with bilateral acetabular dysplasia and coxa valga before (**A**) and after (**B**) Dega osteotomy and proximal femoral varus derotation osteotomy of both hips. A Tutobone^®^ block (**C**) was cut into a triangular shape (**D**) and inserted to stabilize the new acetabular position.

A modification of the original Dega pelvic osteotomy (Hamburg Dega osteotomy) was introduced to our institution by the senior author (R.S.) in 1998. The modified Dega osteotomy was performed under general anaesthesia with the patient supine on a radiolucent table. Salter’s oblique inguinal incision was used starting 1 cm inferior and 4 cm posterior to the anterior superior iliac spine in all cases. The abductor muscles and the tensor muscle were detached from the lateral wall of the ilium to expose the sciatic notch. The cartilaginous apophysis was not split but displaced medially. Two parallel K-wires were inserted into the lateral aspect of the ilium about one centimetre proximal to the acetabular edge and directed to the most medial border of the triradiate cartilage. The cut was then performed with straight osteotomes and extended medially into the medial cortex of the ilium and posteriorly into the sciatic notch leaving a portion of the ilium next to the triradiate cartilage intact as a hinge. An osteotome or laminar spreaders were used to gently open the osteotomy in a controlled manner until sufficient coverage of the femoral head was achieved as determined by fluoroscopy. The graft size was determined by measuring the corresponding gap. A Tutobone^®^ block was fitted and impacted. An internal fixation of the xenograft was not used (Figure 1). 

Depending on patients age and underlying medical conditions either an individual foam block (*n* = 55), a bilateral long leg hip spica cast with an abduction bar (*n* = 37), or an abduction brace (*n* = 9) was applied after surgery to maintain moderate hip abduction for 6 weeks. Patients who received a foam block or abduction brace were allowed to flex the hip up to 90° immediately after surgery. Sitting was allowed three times a day for a maximum of one hour each day when patients reached 90° of hip flexion. After 6 weeks patients returned to a local rehabilitation center in order to start weight-bearing exercises depending on their conditions. These exercises included at least mobilization in a standing frame. 

### 2.4. Radiological Assessment

**Figure 2:**
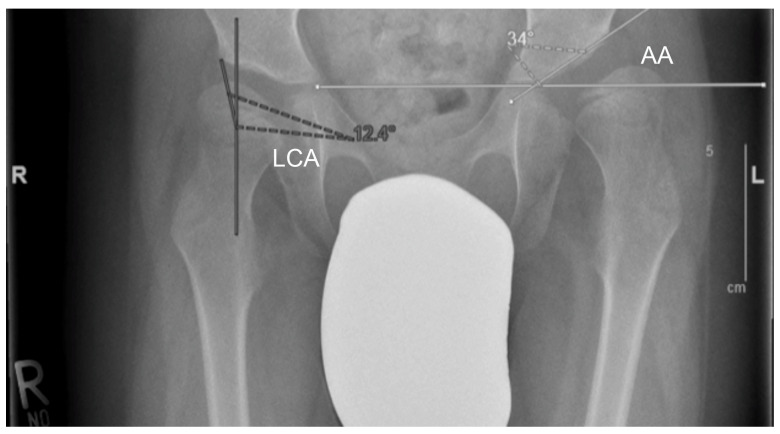
Anteroposterior radiographs of the pelvis were used to measure the lateral centre-edge-angle (LCA) and the acetabular angle of Hilgenreiner (AA). To measure the LCA, a best fitting circle inside the femoral head was drawn. The angle was then measured between two lines from the centre of this circle, one vertically and the other one to the lateral acetabular edge. The AA was measured by using the angle between the Hilgenreiner’s line (line through both triradiate cartilage) and the acetabular roof.

To quantify the results of Dega osteotomy, the acetabular angle of Hilgenreiner (AA) as well as the lateral centre-edge angle (LCA) were analysed on anteroposterior radiographs of the pelvis (Figure 2). 

The normal value of the acetabular angle of Hilgenreiner is 30° at birth. The AA decreases to 15 ± 5.5° during the first 4 years of life and then it remains stable until full hip ossification at maturity. Special values of the AA with regard to the exact patient age are listed in corresponding tables, for example, by Tönnis and Bunken [14,15,16]. The centre-edge angle was originally described by Wiberg in 1939 [17]. In children (3–17 years) an angle of more than 20° can be considered as normal and an angle below 15° as pathological [18]. 

Measurements were performed with Centricity Universal Viewer Zero Footprint^®^ (GE Healthcare, Chicago, IL, USA). Tutobone block incorporation was quantified by using a radiographic scoring system as described by Goldberg [19,20] at the last follow-up. Therefore, the graft appearance, and the bony union at the proximal and distal end were assessed. Regarding graft appearance the score was 0 for resorbed, 1 for mostly resorbed, 2 for largely intact, and 3 for reorganizing. For bony union at the proximal and distal end the score was 0 for non-union, 1 for possible union, and 2 for complete union. All together a maximum of 7 points could be reached indicating excellent graft reorganization and radiographic union (Table 1). 

Two independent blinded orthopaedic surgeons measured the radiographic indices for all pre- and postoperative hip X-rays. Differences in assessment were discussed until consensus was reached.

### 2.5. Statistical Analyses

Descriptive baseline characteristics are reported as mean (± standard deviation (SD)) or as frequencies and percentages, when appropriate. Two measured values were analysed regarding their change over time using linear mixed models: AA and LCA. All models were adjusted for the respective baseline measurement, age at the time of surgery, gender, time between measurements, side (right/left), underlying disease and surgical procedure. To take the cluster structure of the data into account, induced by repeated measurements on patients, patient was included as a random effect. A two-tailed *p*-value < 0.05 was considered to be statistically significant. All analyses were performed using STATA 16.1 (STATA Corporation).

## 3. Results

A total of 147 hips (101 patients) treated by Dega osteotomy (46 bilateral, 55 unilateral) were included in this study. Most of the children had (1) neurological disorders (56% of treated hips) like cerebral palsy (47 patients/71 hips) and myelomeningocele (10 patients/15 hips), (2) congenital hip dysplasia (25 patients/33 hips) and (3) syndrome disorders (15 patients/22 hips). A total of 4 patients (6 hips) were not assigned to one of the groups. Two of these patients suffered from myopathy, one from mucopolysaccharidosis and one patient had a hip joint empyema in early childhood.

The study cohort included 55 female and 46 male patients with a mean age of 5.7 years (SD: 2.86; range: 1.3–12.8) and a body weight of 17.9 kg (SD: 6.71; range: 8.0–44.0) at the time of surgery. The mean thickness of the implanted tutobone^®^ block was 1.3 cm (SD: 0.30; range: 0.6–2.0). 54 patients had an additional proximal femoral varus derotation osteotomy through a separate lateral approach (25 unilateral and 29 bilateral) and 21 patients (22 hips) had open reduction of hip dislocation in addition to Dega osteotomy. 

Final (1-year control) follow-up was at a mean of 16.3 months (SD: 6.87; range 8–44 months). This range occurred due to the fact that some patients represented earlier or later to the regular follow-up examination after 1-year at the outpatient clinic. The minimum follow-up was set at 8 months because all patients had at least 6 months of full-weight bearing with maximum loading of the graft. 

**Figure 3:**
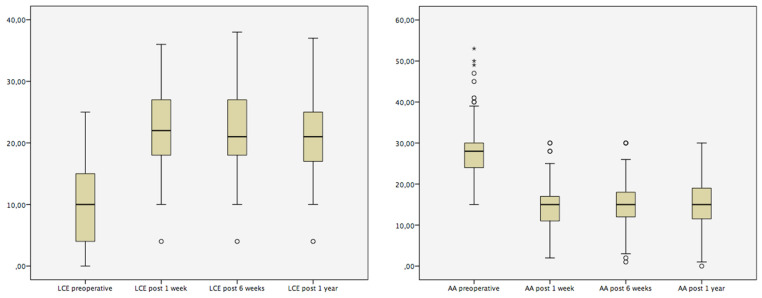
Pre- and postoperative radiographic measurements of the lateral center-edge-angle (LCE) and the acetabular angle (AA) visualized by using boxplots. Circles (°) and asterisks (*) denote weak or strong outliers.

The mean preoperative AA was 28.1 (SD: 6.7), which improved significantly to 14.7 (SD: 5.1) after Dega osteotomy (*p* < 0.001). The AA remained stable at the 6 weeks (mean: 14.9; SD: 5.2; *p* = 0.67) and at the 1-year (mean: 15.2; SD: 5.8; *p* = 0.90) follow-up examinations. The mean LCA was 9.9 (SD: 6.7), which improved significantly to 21.8 (SD: 6.8) after surgery (*p* < 0.001). The LCA remained stable at the 6 weeks (mean: 21.8; SD: 6.4; *p* = 0.92) and at the 1-year (mean: 20.8; SD: 6.9; *p* = 0.62) follow-up examinations as well (Table 2; Figure 3).

Besides, there was no significant difference in the correction rate of the AA and the LCA with regard to the underlying disease using a linear mixed model analysis. In addition, the AA and the LCA were not significantly affected by gender or patient age at surgery.

**Figure 4:**
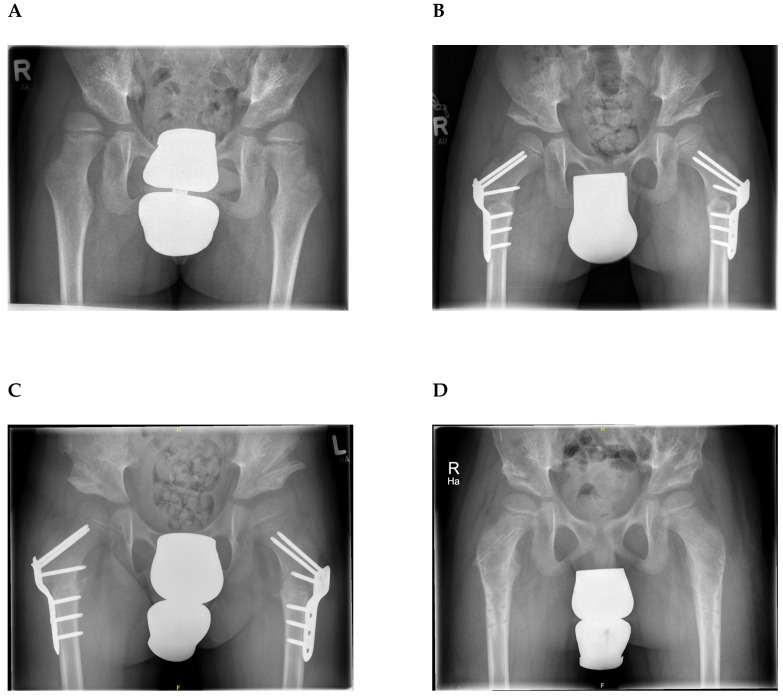
(**A**) Preoperative anteroposterior radiograph of the pelvis of a 6-year-old boy with hip dislocation and acetabular dysplasia on both sides. (**B**) Postoperative radiograph one week after bilateral proximal femoral varus derotation osteotomy and Dega osteotomy using Tutobone^®^ shows a significant improvement of acetabular inclination and coverage of the femoral head on both sides. (**C**) The position of the Tutobone^®^ block as well as acetabular indices remained stable after 6 weeks of immobilization (**D**) The correction of acetabular dysplasia remained stable at the 1-year follow-up examination and the Tutobone^®^ block was incorporated (Goldberg score 6 on the right and 7 on the left side). The femoral plates had been removed one year after surgery.

The mean Goldberg score was 6.6 (SD: 0.6; range 5–7) at the last follow-up. Bone graft displacement or resorption was not detected. Heterotopic ossification occurred in one hip without clinical relevance. There were no graft-related complications during or after surgery like infection, relevant hematoma or pathological fractures. Femoral head necrosis occurred in one hip after open reduction of hip dislocation but recovered without additional treatment (Figure 4).

## 4. Discussion

The use of a bovine xenogeneic bone graft (Tutobone^®^) for Dega osteotomy was safe and effective in the treatment of acetabular dysplasia in children. In this study, with 147 treated hips, the acetabular angle of Hilgenreiner and the lateral centre-edge-angle improved significantly after surgery. Although patients were allowed full-weight bearing 6 weeks after Dega osteotomy both acetabular indices remained stable at the 6 weeks and 1-year follow-up examinations. Besides, excellent bone graft incorporation occurred at the last follow-up with a mean Goldberg score of 6.6 (maximum attainable score: 7). The results of Tutobone^®^ use in orthopaedic and trauma surgery are controversial and most of the studies included a limited number of patients. Patil et al. found an increased nonunion rate (89%) with persisting pain 12 months after tutobone application for subtalar fusion in 9 patients [21]. In contrast, the results of Tutobone^®^ application were promising in the study of Makridis et al. They investigated the incorporation characteristics of Tutobone^®^ in 16 adult patients for reconstruction of iliac crest defects post-harvesting tri-cortical iliac crest bone grafts for pubic symphysis fusion. Integration was defined by the authors as bridging of the interface between the Tutobone^®^ block and the native bone by bone or obliteration of the graft interface. An integration rate of 94 % over a median period of 3 months (range: 2–6) was reported [22]. Meyer et al. revealed a sufficient incorporation of Tutobone^®^ without significant graft sintering or a high infection rate in 9 patients who had either high tibial opening wedge osteotomy or hip arthroplasty [13]. In a current study of 2019 Ibrahim et al. analyzed the outcome after Dega osteotomy in walking children with developmental dysplasia of the hip [11]. They used a Tutobone^®^ block in 12 out of 39 treated hips and reported satisfactory clinical and radiological results after a mean of 33.6 months including all patients. However, conclusions have to be drawn with caution, since the authors did not provide a subgroup analysis of patients with Tutobone^®^ application.

As Tutobone^®^ is a xenograft which is currently certified for oral and maxillofacial surgery and is in off-label use for other operations some concerns about graft rejection and the risk of transmission of infectious disease have to be discussed [23,24]. Due to an elution and sterilisation process prior to application, the immunogenic potential is minimized but the osteoconductive properties are also reduced [21]. Besides an excellent osseointegration, no graft-related complications during or after surgery like infection, relevant hematoma or pathological fracture occurred in our study. In one case, a heterotopic ossification was noted without clinical relevance.

Including 101 patients with 147 treated hips, we report one of the largest studies focusing on the outcome of Dega osteotomy in children with hip dysplasia. In most of the previous studies femoral autograft or iliac crest autograft were used as interposition material for Dega osteotomy. El-Sayed et al. found a significant improvement of the acetabular index in 58 treated hips of children with developmental dysplasia by using either a tricortical iliac crest autograft or a femoral autograft [25]. The mean acetabular index improved from 39° to 18° immediately after Dega osteotomy and was 25° at long-term follow-up, representing some loss of correction [25]. Sung et al. investigated the outcome of Dega pelvic osteotomy using iliac crest allograft in patients with cerebral palsy (150 hips). The mean acetabular index improved from 32.2° to 13.6° after surgery and remained stable with 13.8° at the last follow-up (mean: 2.9 years) [20]. The Goldberg score was <6 in 6% of the treated hips 1 year after surgery [20]. A total of 7% of the treated hips (10 out of 147) had a Goldberg score <6 in our study, but none had a score < 5, indicating excellent graft incorporation. Cost analysis of different bone substitutes used at our institution revealed that an iliac crest allograft block of the same size is more expensive (3-fold) as compared to the bovine xenogeneic bone graft (Tutobone^®^). The use of iliac crest autograft for Dega osteotomy may cause negative side effects like growth disturbance, increased blood loss and an increased operative time, which increases the costs of surgery as well [6,7]. 

Cerebral palsy often is associated with osteopenia [26]. There are different factors related to low bone mineral density in children with cerebral palsy like low nutritional status, low calcium and vitamin D level, physical disability, immobilization and the use of anticonvulsants [5]. The combination of low bone mineral density and muscle imbalance or joint contractures leads to a high fracture risk and an increased risk for delayed union [5,20]. In this study, we found no significant differences in outcome parameters between patients with different underlying disease (3 subgroups analyzed). Patients with cerebral palsy, the largest subgroup (47%) in this series, showed similar results after Dega osteotomy over time as compared to the other groups. 

The limitations of this study include (1) its retrospective design, (2) the lack of a control group with application of autograft or allograft for Dega osteotomy, and (3) the fact that patients were not examined until they reached skeletal maturity. However, bone graft incorporation was excellent at the final follow-up examination 1 year after Dega osteotomy and the correction of acetabular indices remained stable during that time in this study. 

## 5. Conclusions

Dega osteotomy using Tutobone^®^ is safe and effective in the treatment of acetabular dysplasia in children. Besides, excellent bone graft incorporation, the acetabular angle of Hilgenreiner and the lateral centre-edge angle improved significantly after surgery and they remained stable over time. None of the patients developed graft-related complications. Therefore, we recommend the use of Tutobone^®^ for Dega pelvic osteotomy in children with acetabular dysplasia independent of the underlying disease.

## Figures and Tables

**Table 1:** Goldberg radiographic graft scoring system.

1. Graft appearance	
Resorbed	0
Mostly resorbed	1
Largely intact	2
Reorganizing	3
2. Bony union (proximal)	
Non-union	0
Possible union	1
Radiographic union	2
3. Bony union (distal)	
Non-union	0
Possible union	1
Radiographic union	2

**Table 2:** Pre- and postoperative radiographic measurements.

		Acetabular Angle			Lateral Centre-Edge-Angle		
		Right	Left	Both	Right	Left	Both
Preoperative	Mean ± SD	27.2 ± 5.4	28.9 ± 7.6	28.1 ± 6.7	11.2 ± 6.8	8.6 ± 6.4	9.9 ± 6.7
Postoperative (1 week)	Mean ± SD	14.0 ± 5.3	15.4 ± 4.9	14.7 ± 5.1	22.9 ± 7.0	20.4 ± 6.4	21.8 ± 6.8
Postoperative (6 weeks)	Mean ± SD	14.5 ± 5.5	15.4 ± 4.9	14.9 ± 5.2	22.2 ± 6.6	21.3 ± 6.2	21.8 ± 6.4
Postoperative (1 year)	Mean ± SD	14.8 ± 5.9	15.6 ± 5.6	15.2 ± 5.8	21.1 ± 6.7	20.5 ± 7.3	20.8 ± 6.9
